# Altered oxidative stress and antioxidant biomarkers concentrations in pregnant individuals exposed to oil and gas sites in Northeastern British Columbia

**DOI:** 10.1093/toxsci/kfae080

**Published:** 2024-06-19

**Authors:** Matthew W Day, Coreen Daley, Yifan Wu, Maduomethaa Pathmaraj, Marc-André Verner, Élyse Caron-Beaudoin

**Affiliations:** Department of Physical and Environmental Sciences, University of Toronto Scarborough, Scarborough, ON M1C 1A4, Canada; Department of Physical and Environmental Sciences, University of Toronto Scarborough, Scarborough, ON M1C 1A4, Canada; Department of Physical and Environmental Sciences, University of Toronto Scarborough, Scarborough, ON M1C 1A4, Canada; Department of Health and Society, University of Toronto Scarborough, Scarborough, ON M1C 1A4, Canada; Department of Occupational and Environmental Health, School of Public Health, Université de Montréal, Montreal, QC H3C 3J7, Canada; Centre de Recherche en santé Publique, Université de Montréal et CIUSSS du Centre-Sud-de-l'Île-de-Montréal, Montreal, QC H3C 3J7, Canada; Department of Physical and Environmental Sciences, University of Toronto Scarborough, Scarborough, ON M1C 1A4, Canada; Department of Health and Society, University of Toronto Scarborough, Scarborough, ON M1C 1A4, Canada; Dalla Lana School of Public Health, University of Toronto, Toronto, ON M5T 3M7, Canada; Department of Pharmacology & Toxicology, University of Toronto, Toronto, ON M5G 2C8, Canada

**Keywords:** volatile organic compounds, oil and gas wells, unconventional oil and gas, oxidative stress, antioxidant activity

## Abstract

Northeastern British Columbia is a region of prolific unconventional oil and gas (UOG) activity. UOG activity can release volatile organic compounds (VOCs) which can elevate oxidative stress and disrupt antioxidant activity in exposed pregnant individuals, potentially increasing the risk of adverse pregnancy outcomes. This study measured biomarkers of oxidative stress and antioxidant activity in pooled urine samples of 85 pregnant individuals living in Northeastern British Columbia, to analyze associations between indoor air VOCs, oil and gas well density and proximity metrics, and biomarker concentrations. Concentrations of catalase, superoxide dismutase (SOD), glutathione S-transferase, total antioxidant capacity, 6-hydroxymelatonin sulfate (aMT6s), malondialdehyde (MDA), 8-hydroxy-2′-deoxyguanosine (8-OHdG), and 8-isoprostane were measured using assay kits. Associations between exposure metrics and biomarker concentrations were determined using multiple linear regression models adjusted for biomarker-specific covariables. UOG proximity was associated with decreased SOD and 8-OHdG. Decreased 8-OHdG was associated with increased proximity to all wells. Decreased aMT6s were observed with increased indoor air hexanal concentrations. MDA was negatively associated with indoor air 1,4-dioxane concentrations. No statistically significant associations were found between other biomarkers and exposure metrics. Although some associations linked oil and gas activity to altered oxidative stress and antioxidant activity, the possibility of chance findings due to the large number of tests cannot be discounted. This study shows that living near UOG wells may alter oxidative stress and antioxidant activity in pregnant individuals. More research is needed to elucidate underlying mechanisms and to what degree UOG activity affects oxidative stress and antioxidant activity.

Unconventional oil and gas (UOG) production is a common practice where unconventional reservoirs can only be exploited through stimulating techniques like hydraulic fracturing (US EPA 2013). Hydraulic fracturing involves pumping fracturing fluid consisting of water, proppant, and other chemical additives at high pressure to fracture targeted rock formations (US EPA 2013). Once fractured, the internal pressure of the rock formation drives hydraulic fracturing fluids and produced water back up the wellbore to be disposed (Hecobian et al. 2019). If improperly disposed, the flowback can create environmental hazards including the release of volatile organic compounds (VOCs) (Gilman et al. 2013; Srebotnjak and Rotkin-Ellman 2014; Chittick and Srebotnjak 2017). Fugitive emissions of VOCs can also originate from leaks in midstream production structures, condensate tanks, pipelines, flaring, and transportation (Adgate et al. 2014; Varona-Torres et al. 2017).

Northeastern British Columbia sits on an important source of natural gas, the Montney Formation (Rogers et al. 2014). It is therefore a region of extensive oil and gas exploitation with approximately 35,000 wells drilled so far (Caron-Beaudoin et al. 2023) (Fig. 1). Since the early 2000s, UOG wells have become the norm, vastly overtaking conventional wells as the predominant type of newly drilled wells (Adams et al. 2016). This expanding industry and its emissions could lead to increased exposure to contaminants like VOCs in nearby populations, including vulnerable subgroups like pregnant women.

**Fig. 1.**
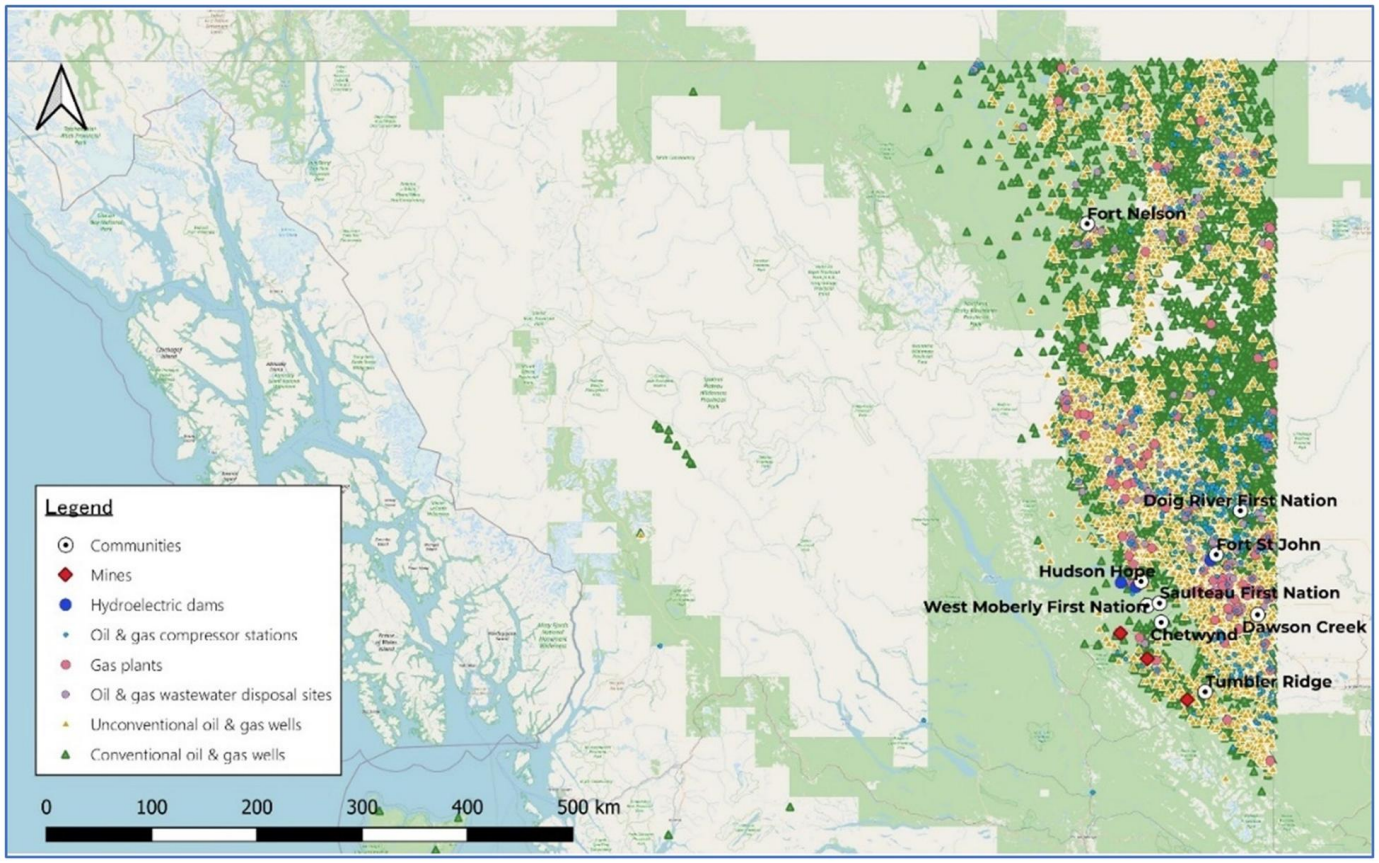
Oil and gas wells in Northeastern British Columbia, Canada. Location of mines, hydroelectric dams, and oil and gas operations (wells, compressor stations, gas plant, wastewater disposal sites) in Northeastern British Columbia, Canada.

Exposure to VOCs during pregnancy has been associated with reduced birth length, head circumference, birth weight, and an increased incidence of preterm birth (Chen et al. 2000; Llop et al. 2010; Zahran et al. 2012; Bergstra et al. 2021). Epidemiological studies have shown increased rates of negative birth outcomes, including reduced birth weight, preterm birth, and congenital malformations in correlation with UOG well proximity and density (Stacy et al. 2015; Casey et al. 2016; Currie et al. 2017; Walker Whitworth et al. 2018; Caron-Beaudoin et al. 2021). Previous studies by our group in Northeastern British Columbia have reported increased concentrations of some VOCs in indoor air and tap water, as well as elevated concentrations of some VOC metabolites in pregnant individuals (Caron-Beaudoin et al. 2018, 2022). Individuals living in this region could therefore be at higher risk for adverse health outcomes due to exposure to environmental pollutants released from nearby UOG sites.

Exposure to pollutants can create oxidative stress by increasing the production of reactive oxygen species (ROS) resulting in lipid peroxidation, oxidation of proteins, and DNA damage (Kiruthiga et al. 2007). Urinary biomarkers have been established to assess the impacts of ROS. For example, lipid peroxidation produces malondialdehyde (MDA) and 8-Isoprostanes (8-IP), whereas DNA oxidation produces 8-hydroxydeoxyguanosine (8-OHdG) (Draper et al. 1984; Wu et al. 2004; Graille et al. 2020). Antioxidant enzymes like superoxide dismutase (SOD), catalase (CAT), and glutathione S-transferase (GST) act to reduce concentrations of common ROS species (Bandyopadhyay et al. 1999; Röth et al. 2011). Altered urinary concentrations of antioxidant enzymes can be an indicator of elevated oxidative stress. Whether antioxidant concentrations increase or decrease depends on the severity of oxidative stress (Xiao et al. 2003; Myatt and Cui 2004; Faraonio et al. 2006; Liu et al. 2008). Increased oxidative stress has been linked to the pathogenesis of most diseases and has been associated with negative birth outcomes (Tabassum and Jeong 2019; Toboła-Wróbel et al. 2020; Yaribeygi et al. 2020; Forman and Zhang 2021).

In 2019, the Exposures in the Peace River Valley (EXPERIVA) study was undertaken to measure exposure to VOCs and trace elements in a cohort of pregnant individuals living in Northeastern British Columbia. Higher concentrations of some VOCs in indoor air and tap water were correlated with the density and proximity of oil and gas wells (Caron-Beaudoin et al. 2022). Using birth records data over a period of 10 yr in the same region, associations between oil and gas activity and preterm birth, low birthweight and maternal depression were observed (Caron-Beaudoin et al. 2021; Aker et al. 2022). Although oxidative stress during pregnancy can lead to negative birth outcomes (Kim et al. 2005; Toboła-Wróbel et al. 2020), its role as a mediating mechanism remains unknown in the context of UOG. In this study, we aimed to: (i) measure oxidative stress and antioxidant activity biomarkers in urine samples from EXPERIVA participants; (ii) determine associations between density/proximity of oil and gas wells, indoor air VOC concentrations, and urinary biomarkers concentrations. It is expected that moderate oxidative stress will result in increased antioxidant activity and high oxidative stress would result in a decreased antioxidant response.

## Materials and methods

### Study area and recruitment

More details on the EXPERIVA study recruitment are published elsewhere (Caron-Beaudoin et al. 2022). Briefly, 92 pregnant individuals living in Northeastern British Columbia were recruited for this study from May to September 2019, 7 of which did not complete sample collection. Recruitment took place in 4 midwifery and medical clinics located in Chetwynd and Dawson Creek. During their prenatal care visit, interested and eligible participants (≥18 yr, English speaking) were met privately by a member of the research team and received information about the research project. Pregnant individuals wanting to participate signed a consent form and received all the material needed to collect indoor air and urine samples at home. Participants also filled out a questionnaire on sociodemographic, housing, and physiological characteristics (e.g. weight, height, gestational week), and sources of VOCs (e.g. smoking). The EXPERIVA study was approved by the Northern Health Research Review Committee and by the Université de Montréal Institutional Review Board (#CERC-18-003-P). The West Moberly First Nations, Saulteau First Nations, and Treaty 8 Tribal Association provided informed consent prior to recruitment. Collected samples are considered “on loan” for the duration of the research to respect cultural and spiritual traditions.

### Urine samples

Daily spot urine samples were collected at home by each participant. Participants were instructed to provide 7 urine samples collected over 7 consecutive days. Samples were collected at night with a median collection time of 9:30 PM. All urine samples were stored frozen at −20 °C by the participant until retrieval by a member of our research team. Samples were transported on ice to the laboratory for analysis.

### Biomarker assay kits

For the biomarker analyses, urine samples were pooled for each participant, then centrifuged to collect the supernatant to improve the consistency of assay detection. To quantify the degree of oxidative stress and the accompanying antioxidant response, a number of biomarkers were measured in pooled urine samples using assay kits. MDA and 8-IP were used as biomarkers of lipid peroxidation while 8-OHdG was used as a biomarker of DNA oxidation. CAT, SOD, GST, TAC, and aMT6s were measured to assess antioxidant activity. These antioxidant enzymes were chosen as biomarkers of antioxidant activity because they act on the most common ROS species, are present in high concentrations in the antioxidant response, and are detectable in urine (Perrin-Nadif et al. 1996; Yang et al. 2013; Peluso and Raguzzini 2016). Melatonin, in addition to having its own antioxidant properties, upregulates the nuclear factor erythroid 2-related factor 2 (Nrf2) antioxidant pathway in a dose-dependent manner (Chen et al. 2017). Melatonin is of particular importance as the Nrf2 pathway transcribes antioxidant enzymes like CAT, SOD, and GST (Miller et al. 2013; Guo et al. 2017; Yu et al. 2019; Morvaridzadeh et al. 2020). Circulating melatonin is metabolized in the liver before excretion in urine and therefore is not present in high enough concentrations to be detectable in urine. Urinary melatonin sulfate (aMT6s) was measured instead because it directly correlates with plasma melatonin concentrations (Bojkowski et al. 1987; Graham et al. 1998; Braam and Spruyt 2022). Braam and Spruyt (2022) performed an analysis of 78 men and determined urinary aMT6s accounted for 72% of the variation in total plasma melatonin and peaks in total plasma melatonin were well represented by urinary aMT6s. Another study determined urinary aMT6s accounted for 75% of the variation in plasma melatonin over a 24-h period (Bojkowski et al. 1987).

Various assay kits were used to measure concentrations of urinary MDA (Abcam #ab233471), 8-IP (Abcam #ab175819), 8-OHdG (Abcam #ab201734), SOD (Abcam #ab65354), CAT (Abcam #ab83464), GST (Abcam # ab65326), TAC (Cayman #709001), and aMT6s (IBL/TECAN #RE54031), following the manufacturers’ instructions. Each assay kit had a unique methodology but, in all kits, standards, and samples were measured in duplicate.

### Exposure metrics

Phase-specific density and inverse distance weighting squared (ID^2^W) metrics for both conventional and UOG wells were calculated. To calculate these metrics for each participant, data on 35,360 oil and gas wells were collected from the British Columbia Energy Regulator. ID^2^W metrics are calculated using a weighted average with closer wells receiving a higher weight. ID^2^W metrics were used rather than inverse distance weighting (IDW) metrics because it better represents how exposure potential declines rapidly with distance, as a function of the distance squared (Rasmussen et al. 2016). Full explanations of ID^2^W calculations are detailed elsewhere (Caron-Beaudoin et al. 2023). Combined metrics included both conventional and unconventional wells and were calculated with no buffer, 10, and 5 km buffer zones around the participant’s residence. Separate metrics for conventional and UOG wells were calculated with these same buffers. Additionally, phase-specific UOG metrics were calculated for each phase of the unconventional extraction process including pad preparation, drilling, stimulation, and production using no buffer, 5 and 10 km buffer zones.

Details on the 47 VOCs measured in indoor air samples are available elsewhere (Caron-Beaudoin et al. 2022). Of the 47 analyzed VOCs, only those linked to increase oxidative stress in the scientific literature and with a high proportion of EXPERIVA participants with indoor air concentrations above the Canadian Health Measure Survey 95th percentiles were selected for further statistical analyses (Caron-Beaudoin et al. 2022). The VOCs matching these requirements included acetone (Armutcu et al. 2005; Long et al. 2021), chloroform (Beddowes et al. 2003; Santos et al. 2017), 1,4-dioxane (Meng 2018; Chen et al. 2022), decanal (Hong et al. 2009; Maskrey et al. 2016), hexanal (Hoelzer et al. 2016; Li et al. 2021), xylene (McKenzie et al. 2012; Salimi et al. 2017), and styrene (Röder-Stolinski et al. 2008; McKenzie et al. 2012; Dreisbach 2013). Benzene, toluene, ethylbenzene, and xylene (BTEX) are a subgroup of VOCs which are often used to estimate air quality and overall concentrations of VOCs (Yadav and Pandey 2018). A BTEX metric of the summed concentrations of benzene, toluene, ethylbenzene, and xylene was included in our analysis as BTEX exposure has previously been linked to increased oxidative stress and altered antioxidant activity in previous studies (Xiong et al. 2016; Rafiee et al. 2022).

### Statistical analysis

Biomarker concentrations below the limits of detection were given values half the limit of detection, and correlations between biomarkers were determined using Spearman’s rank correlation. Previous studies from this cohort have found significantly higher indoor air VOCs in Indigenous participants compared with nonindigenous participants (Caron-Beaudoin et al. 2022) and therefore differences in biomarker concentrations between these subgroups were analyzed using a nonparametric Kruskal–Wallis test. The Kruskal–Wallis test was chosen because the assumptions for an ANOVA were violated as residuals were not normally distributed.

Multiple linear regression analysis was then performed to evaluate associations between exposure metrics and urinary biomarker concentrations, expressed as standardized betas. Multiple linear regression analyses were performed in R studio (version 4.2.2.). Log10-transformed urinary biomarkers concentrations were used to ensure that residuals were normally distributed. The multicollinearity using the variance inflation factor was verified for each model. Covariables for multiple linear regression analysis were selected a priori on previous literature on predictors of increased proximity to oil and gas operations, increased levels of VOCs in indoor air, and disruption of ROS or antioxidant activity. Common to all biomarkers were participant age, gestational week at the time of recruitment, smoking status, indigenous status, and urinary creatinine. Increased age has previously been linked to increased urinary biomarkers of oxidative stress (Sakano et al. 2009; Mukli et al. 2022). Smoking is strongly associated with urinary concentrations of oxidative stress biomarkers where smokers show elevated urinary concentrations compared with nonsmokers (Seet et al. 2011; Mesaros et al. 2012; Joshi et al. 2020). The gestational week was included as a covariable as blood concentrations of oxidative stress biomarkers have been shown to change over the course of a pregnancy, often peaking in the second trimester (Uotila et al. 1991; Casanueva and Viteri 2003; Hung et al. 2010) and antioxidant activity has been shown to increase throughout a pregnancy before a rapid drop before birth (Toboła-Wróbel et al. 2020). Urinary creatinine was included as it can act as a baseline for urinary dilution and helps to standardize assay results (Abuawad et al. 2022). Sample collection time was included as a binary covariable for aMT6s analysis due to its diurnal cycle. This binary variable indicates a sample time before or after 8 PM due to the rapid increase of melatonin secretion around this time (Liu et al. 2000).

## Results

### Characteristics of EXPERIVA participants

The 85 participants had a median age of 29 yr (range: 18 to 40) at the time of sampling and a median gestational age of 23 wk (range: 7 to 39). Only 8% (*n* = 7) were smokers at the time of recruitment with the remainder having never smoked or quit smoking before their pregnancy (Table 1).

**Table 1.** Characteristics of pregnant individuals from the EXPERIVA study.

	All participants (*n* = 85)	Indigenous (*n* = 15)	Nonindigenous (*n* = 70)
Characteristic	Median (min; max)	Median (min; max)	Median (min; max)
Age (yr)	29 (18; 40)	28 (18; 40)	29 (20; 38)
Gestational age (wk)	23 (7; 39)	31 (7; 37)	23 (7; 39)
Smoker at the time of recruitment	*n* (%)	*n* (%)	*n* (%)
Yes	7 (8)	2 (14)	5 (7)
No	78 (92)	13 (87)	65 (93)
Number of wells (all)	Median (min; max)	Median (min; max)	Median (min; max)
10 km	90 (0; 346)	89 (1; 346)	91 (0; 331)
5 km	9 (0; 138)	18 (0; 112)	8 (0; 138)
Number of wells (conventional)			
10 km	46 (1; 326)	49 (3; 262)	45 (1; 326)
5 km	10 (0; 49)	10 (0; 40)	10 (0; 49)
Number of wells (unconventional)			
10 km	160 (0; 578)	153 (0; 578)	165 (0; 448)
5 km	12 (0; 210)	15 (0; 194)	12 (0; 210)
ID^2^W (all)			
No buffer	12.02 (2.23; 101.78)	12.16 (2.51; 69.52)	12.02 (2.23; 101.78)
10 km	4.06 (0.01; 97.56)	4.47 (0.03; 60.19)	3.89 (0.01; 97.56)
5 km	1.64 (0; 93.07)	1.98 (0; 53.47)	1.62 (0; 93.07)
ID^2^W (conventional)			
No buffer	4.13 (1.35; 46.65)	4.25 (1.48; 33.35)	4.13 (1.35; 46.65)
10 km	1.15 (0.01; 40.07)	1.24 (0.03; 29.71)	1.08 (0.01; 40.07)
5 km	0.59 (0; 37.47)	0.67 (0.13; 27.78)	0.55 (0; 37.47)
ID^2^W (unconventional)			
No buffer	7.42 (0.88; 90.09)	7.48 (1.00; 36.16)	7.42 (0.88; 90.09)
10 km	2.57 (0; 87.73)	2.61 (0; 30.48)	2.56 (0; 87.73)
5 km	0.68 (0; 83.78)	0.98 (0; 25.68)	0.65 (0; 83.78)
Indoor air VOCs (μg/m^3^)			
Acetone	10.4 (0.2; 106)	15.7 (3.1; 33.4)	10.2 (0.2; 22.4)
Chloroform	0.8 (0.045; 4.9)	1.15 (0.05; 4.9)	0.60 (0.045; 4.6)
1,4-Dioxane	0.10 (0.04; 11.5)	0.15 (0.05; 4.5)	0.10 (0.04; 11.5)
Decanal	3.30 (0.1; 9.7)	4.25 (0.5; 6.8)	3.10 (0.1; 9.7)
Hexanal	20.4 (0.1; 157)	18.4 (3.3; 82.1)	20.25 (0.1; 157)
m/p-Xylene	3.00 (0.05; 486)	3.2 (0.4; 486)	3.1 (0.05; 21.8)
o-Xylene	0.90 (0.05; 105)	0.9 (0.2; 105)	0.95 (0.05; 26.8)
Styrene	0.90 (0.05; 17.3)	0.9 (0.2; 17.3)	0.9 (0.05; 1.8)
BTEX	10.6 (0.245; 925.8)	10.7 (2.55; 778)	10.6 (0.245; 925.8)
Covariables			
Urinary creatinine (g/l)	0.94 (0.2; 2.18)	0.93 (0.41; 1.3)	0.94 (0.2; 2.18)

Distributions of age, gestational age (weeks), smoking status and oil and gas wells density and proximity metrics for both Indigenous and nonindigenous participants can be seen in Table 1. The median density of conventional wells within 10 and 5 km of the participants residence was 46 wells (range: 1 to 326) and 10 wells (range: 0 to 46), respectively. The median density of unconventional wells within 10 and 5 km of the participants residence was 160 wells (range: 0 to 578) and 12 wells (range: 0 to 210), respectively. The phase-specific ID^2^W values for UOG wells in pad preparation, drilling, stimulation, and production phases are detailed elsewhere (Caron-Beaudoin et al. 2023). No statistically significant differences were observed between Indigenous and nonindigenous participants in terms of oil and gas wells density and proximity metrics.

Ranges of indoor air concentrations for selected VOCs can be seen in Table 1. It is noteworthy that chloroform concentrations were significantly higher in Indigenous compared with nonindigenous participants as defined by the Mann–Whitney *U* test (Caron-Beaudoin et al. 2022).

### Biomarker concentrations

Concentrations of oxidative stress biomarkers MDA, 8-IP, and 8-OHdG were measured and are presented in Table 2. Concentrations of biomarkers by age groups and trimesters are presented in Table 3, and by smoking status in Table 4. Participants had a median concentration of 24.65 μM MDA (range: 2.04 to 51.20), 0.009 µg/l 8-IP (range: 0.01 to 1.03) and 75.47 ng/ml 8-OHdG (range: 21.31 to 173.49) (Table 2). Nonparametric Kruskal–Wallis tests were performed and no statistically significant differences in biomarker concentrations were found between age groups, trimesters, smokers and nonsmokers, or Indigenous and nonindigenous populations.

**Table 2.** Oxidative stress and antioxidant activity biomarkers concentrations in EXPERIVA study participants.

Biomarker	All participants (*n* = 85)	Indigenous (*n* = 15)	Nonindigenous (*n* = 70)	Detection frequency
	Median (min; max)	Median (min; max)	Median (min; max)	*n* (%)
*Oxidative stress biomarkers*				
MDA (μM)	24.65 (2.04; 51.20)	21.92 (2.04; 42.70)	24.71 (2.82; 51.20)	85 (100)
8-IP (µg/l)	0.09 (0.01; 1.03)	0.06 (0.02; 0.64)	0.10 (0.007; 1.03)	85 (100)
8-OHdG (ng/ml)	75.47 (21.31; 173.49)	75.86 (25.06; 155.51)	75.07 (21.31; 173.49)	85 (100)
*Antioxidant activity biomarkers*				
TAC (mM_Trolox_)	2.92 (0.26; 5.66)	2.97 (0.26; 5.66)	2.88 (0.42; 5.31)	83 (97.6)
SOD (% inhibition)	35.76 (1.54; 99.00)	36.11 (5.00; 99.00)	35.42 (1.54; 99.00)	76 (89.4)
CAT (mU/ml)	0.04 (0.001; 0.19)	0.04 (0.001; 0.12)	0.04 (0.001; 0.19)	76 (89.4)
GST (mU/min/ml)	109.12 (22.7; 246.42)	101.77 (30.29; 246.42)	109.12 (22.70; 241.52)	85 (100)
aMT6s (ng/ml)	231.71 (23.59; 2082.77)	216.00 (68.72; 41.85)	233.74 (23.59; 2082.77)	84 (98.8)

**Table 3.** Oxidative stress and antioxidant activity biomarkers concentrations in EXPERIVA study participants by age and pregnancy trimester.

Biomarker	Ages 18–29 (*n* = 29)	Ages 30–40 (*n* = 56)	First trimester (*n* = 15)	Second trimester (*n* = 39)	Third trimester (*n* = 31)
	Median (min; max)	Median (min; max)	Median (min; max)	Median (min; max)	Median (min; max)
*Oxidative stress biomarkers*					
MDA (μM)	21.37 (2.82; 42.70)	25.40 (2.04; 51.20)	24.11 (7.20; 47.84)	25.58 (4.74; 51.20)	21.92 (2.04; 42.70)
8-IP (µg/l)	0.10 (0.01; 0.64)	0.07 (0.01; 1.03)	0.07 (0.01; 0.42)	0.07 (0.01; 1.03)	0.10 (0.01; 0.66)
8-OHdG (ng/ml)	68.38 (23.47; 150.44)	79.27 (21.31; 173.49)	82.79 (21.31; 128.77)	67.84 (24.31; 173.49)	90.29 (23.47; 155.51)
*Antioxidant activity biomarkers*					
TAC (mM_Trolox_)	2.91 (0.50; 5.14)	2.92 (0.26; 5.66)	2.30 (0.50; 3.61)	3.03 (0.26; 5.31)	2.98 (0.65; 5.66)
SOD (% inhibition)	52.66 (1.54; 99.00)	31.94 (3.03; 99.00)	51.51 (3.03; 99.00)	32.64 (1.54; 99.00)	37.50 (5.00; 99.00)
CAT (mU/ml)	0.04 (0.001; 0.18)	0.03 (0.001; 0.19)	0.04 (0.001; 0.17)	0.03 (0.0001; 0.19)	0.04 (0.001; 0.18)
GST (mU/min/ml)	95.82 (30.29; 217.00)	121.36 (22.70; 246.42)	117.54 (22.70; 246.42)	98.09 (30.29; 224.36)	121.34 (30.29; 241.52)
aMT6s (ng/ml)	226.09 (50.00; 1700.77)	233.62 (23.59; 2082.78)	279.19 (95.91; 1154.23)	231.65 (32.48; 1700.77)	193.43 (23.60; 2082.77)

**Table 4.** Oxidative stress and antioxidant activity biomarkers concentrations in EXPERIVA study participants by smoking status.

Biomarker	Smoker (*n* = 7)	Non-smoker (*n* = 78)
	Median (min; max)	Median (min; max)
*Oxidative stress biomarkers*		
MDA (μM)	24.11 (11.58; 41.85)	24.65 (2.04; 51.20)
8-IP (µg/l)	0.10 (0.01; 0.46)	0.09 (0.01; 1.03)
8-OHdG (ng/ml)	79.75 (65.79; 147.67)	74.62 (21.31; 173.49)
*Antioxidant activity biomarkers*		
TAC (mM_Trolox_)	3.24 (0.26; 4.86)	2.91 (0.42; 5.66)
SOD (% inhibition)	68.05 (29.17; 99.00)	33.68 (1.54; 99.00)
CAT (mU/ml)	0.05 (0.01; 0.11)	0.04 (0.001; 0.19)
GST (mU/min/ml)	79.42 (37.88; 177.77)	110.34 (22.70; 246.42)
aMT6s (ng/ml)	466.13 (109.48; 1338.07)	230.22 (23.59; 2082.77)

**Fig. 2.**
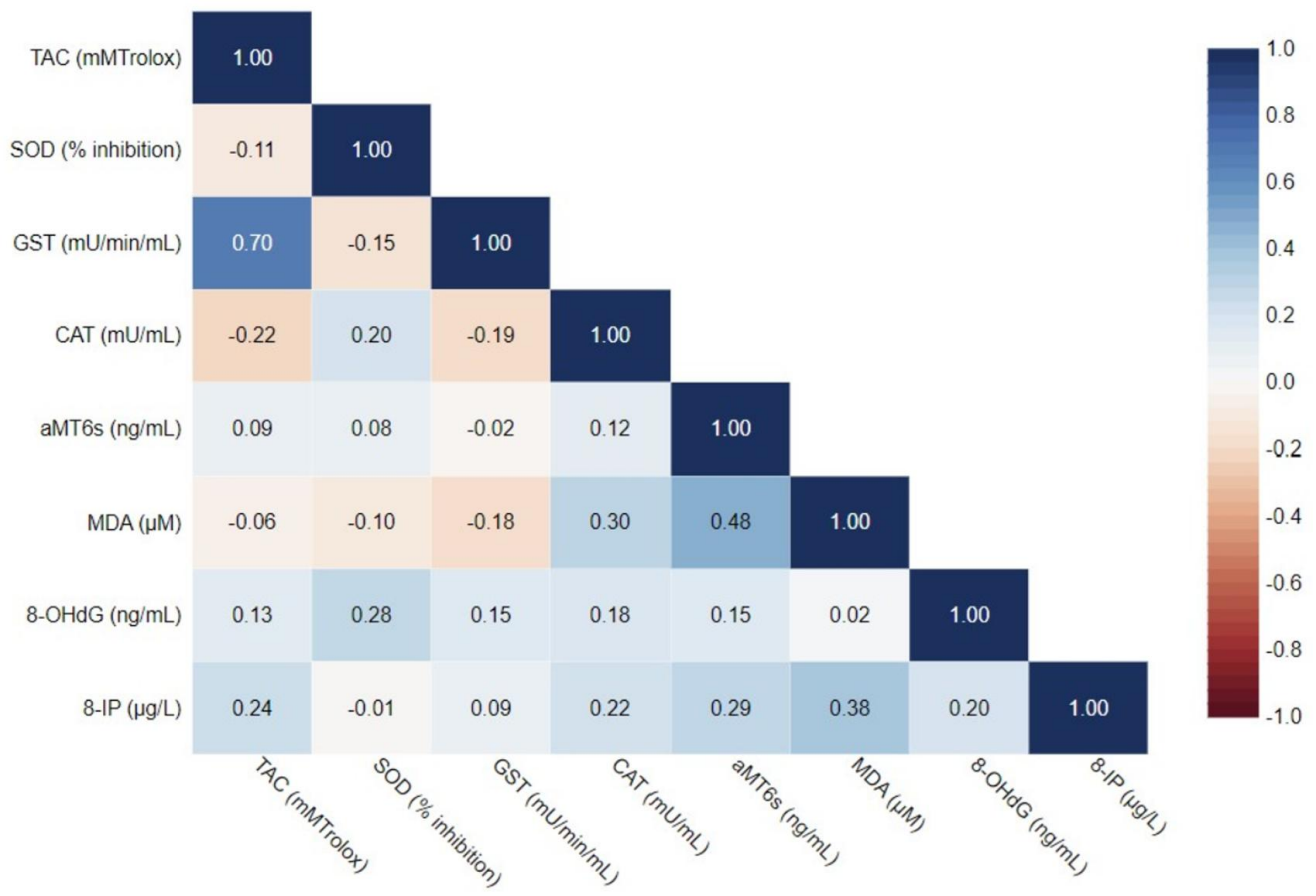
Spearman rank correlations between urinary biomarkers of oxidative stress and antioxidant activity.

Concentrations of antioxidant biomarkers TAC, CAT, SOD, GST, and aMT6s were also measured. Participants had a median concentration of 2.92 mM_Trolox_ TAC (range: 0.26 to 5.66), 0.04 mU/ml CAT (range: 0.001 to 0.19), 109.12 mU/min/ml GST (range: 22.7 to 246.42), 231.7 ng/ml aMT6s (range: 23.59 to 2082.77), and a median 35.76% inhibition of SOD (range: 1.54 to 99.00) (Table 2). Concentrations of antioxidant biomarkers were within the expected range provided with each kit. Correlations between biomarkers of oxidative stress and antioxidant activity are presented in Figure 2. Positive correlations were noted for GST and TAC; MDA and aMT6s; MDA and 8-IP.

### Associations between biomarker concentrations, density/proximity of oil and gas wells, and VOCs

Standardized betas from multiple linear regression models between exposure metrics and biomarker concentrations are presented in Table 5. We observed no clear pattern of association. Where associations were observed, they generally strengthened with decreased buffer size but did not seem to be vary by phase (e.g. drilling, production). Among the few associations that were observed, an increase in ID^2^W of UOG wells at no buffer, 10 and 5 km buffer zones, and an increase in UOG well density at 10 km buffer zones were associated with a decrease in urinary concentrations of SOD. An increase in ID^2^W of all wells within 5 km was positively associated with urinary CAT. An increase in ID^2^W for both all wells and UOG wells at no buffer 10 and 5 km buffer zones was associated with a decrease in urinary 8-OHdG. Increased density of all wells and UOG wells within 5 km was also associated with a decrease in urinary 8-OHdG. Increased density of conventional wells within 5 km was associated with an increase in urinary MDA. An increase in ID^2^W of UOG wells within 5 km was associated with a decrease in urinary aMT6s concentrations.

**Table 5.** Associations (standardized betas) between exposure metrics and log10-transformed oxidative stress and antioxidant biomarkers concentrations in the EXPERIVA study.

Exposure metrics	TAC	SOD	GST	CAT	aMT6s	MDA	8-OHdG	8-IP
All—ID^2^W no buffer	−0.020	−0.134	−0.004	0.144	−0.128	0.092	−0.237*	−0.024
All—ID^2^W 10 km	−0.031	−0.150	−0.020	0.172	−0.142	0.093	−0.236*	−0.014
All—ID^2^W 5 km	−0.036	−0.153	−0.023	**0.182^✝^**	−0.144	0.089	−0.242*	−0.008
All—Density 10 km	0.036	−0.118	0.038	−0.032	−0.049	0.080	−0.103	−0.086
All—Density 5 km	0.018	−0.148	−0.007	0.071	−0.119	0.068	−**0.226^✝^**	−0.080
UOG—ID^2^W no buffer	−0.002	−0.228*	−0.017	0.115	−0.146	0.030	−0.281*	−0.090
UOG—ID^2^W 10 km	−0.0003	−0.224*	−0.028	0.142	−0.163	0.038	−0.290*	−0.074
UOG—ID^2^W 5 km	0.002	−0.218*	−0.031	0.152	−**0.173^✝^**	0.043	−0.299*	−0.066
UOG—Density 10 km	−0.008	−**0.196^✝^**	0.034	−0.052	0.006	0.003	−0.075	−0.114
UOG—Density 5 km	−0.007	−0.184	−0.013	0.063	−0.099	0.019	−**0.205^✝^**	−0.090
UOG—PAD no buffer	0.020	−0.049	0.000	−0.138	0.134	0.022	0.099	−0.060
UOG—PAD 10 km	0.074	−0.015	0.015	−0.097	0.121	0.078	0.107	0.003
UOG—PAD 5 km	0.106	−0.030	0.049	−0.095	0.071	0.077	0.078	−0.011
UOG—DRILL no buffer	−0.024	−0.034	−0.048	−0.134	0.153	0.040	0.062	−0.072
UOG—DRILL 10 km	0.026	0.008	−0.027	−0.097	0.137	0.093	0.083	−0.009
UOG—DRILL 5 km	0.072	−0.008	0.019	−0.097	0.089	0.092	0.047	−0.031
UOG—STIM no buffer	−0.052	−0.035	−0.031	−0.076	0.135	0.084	0.044	−0.074
UOG—STIM 10 km	−0.034	−0.013	−0.021	−0.055	0.113	0.115	0.062	−0.027
UOG—STIM 5 km	−0.025	−0.026	0.008	−0.041	0.078	0.119	0.043	−0.026
UOG—PROD no buffer	0.079	−0.142	0.070	−0.113	0.025	−0.115	0.041	−0.053
UOG—PROD 10 km	0.094	−0.133	0.060	−0.087	0.013	−0.110	0.049	−0.023
UOG—PROD 5 km	0.094	−0.119	0.062	−0.072	−0.013	−0.098	0.041	0.003
Conv—ID^2^W no buffer	−0.035	0.085	0.018	0.104	−0.028	0.132	−0.033	0.090
Conv—ID^2^W 10 km	−0.066	0.063	0.006	0.126	−0.027	0.135	−0.008	0.098
Conv—ID^2^W 5 km	−0.086	0.050	0.006	0.137	−0.011	0.125	−0.002	0.103
Conv—Density 10 km	0.068	0.080	0.014	0.020	−0.094	0.123	−0.061	0.019
Conv—Density 5 km	0.072	0.036	0.014	0.049	−0.107	**0.153^✝^**	−0.144	−0.004
Acetone	0.032	0.085	0.017	0.071	−0.002	0.106	0.062	0.023
Chloroform	−0.159	−0.127	−0.147	−0.106	0.060	−0.017	−0.138	−0.143
1,4-Dioxane	−0.055	0.003	0.074	0.075	0.066	−0.209*	0.185	−0.078
Decanal	0.121	−0.059	0.150	0.078	−0.037	−0.037	−0.182	0.019
Hexanal	0.035	−0.101	0.015	−0.034	−0.249*	−0.049	−0.050	**0.165^✝^**
mp-Xylene	−0.028	0.003	−0.042	0.153	−0.095	0.048	0.021	−0.085
o-Xylene	−0.022	0.002	−0.040	0.148	−0.115	0.039	−0.007	−0.075
Styrene	−0.015	0.025	−0.045	0.057	−0.086	0.085	0.002	−0.102
BTEX	0.054	−0.004	0.062	−0.066	−0.014	0.113	−0.068	−0.097

Sample size = 85. Pooled urine samples run in duplicate for assays. Significance is indicated by *P*-value symbols (*P ≤ 0.05; ^✝^*P* ≤ 0.10). Standardized betas were calculated using multiple linear regression models. TAC, SOD, GST, and CAT models were standardized for age, gestational week at the time of recruitment, smoking status, Indigenous status, and urinary creatinine. aMT6 models were adjusted for age, gestational week at the time of recruitment, smoking status, Indigenous status, urinary creatinine, and time of sample collection. MDA, 8-IP, and 8-OHdG models were adjusted for melatonin, age, gestational week at the time of recruitment, smoking status, Indigenous status and urinary creatinine.

Increased indoor air hexanal concentrations were associated with a decrease in urinary aMT6s and an increase in urinary 8-IP. Increased indoor air 1,4-dioxane concentrations were associated with a significant decrease in urinary MDA. No statistically significant relationships or strong trends were observed between TAC, GST, and any exposure metrics.

## Discussion

Previous studies by our group reported associations between oil and gas wells metrics and increased concentrations of VOCs in indoor air and tap water in EXPERIVA (Caron-Beaudoin et al. 2022). Using birth records data, we also reported nonmonotonic associations between oil and gas well proximity/density metrics and lower birthweight as well as increased odds of preterm birth (Caron-Beaudoin et al. 2021). The results of the current study shed some light on the potential implication of oxidative stress as a mediating mechanism for these associations.

### Antioxidant activity

Some studies suggest that the degree of oxidative stress could be a determining factor in how concentrations of antioxidant biomarkers change in response to exposure to oxidative stress-inducing pollutants (Gechev et al. 2002; Wei and Lee 2002; Rodriguez et al. 2004). In a proteomics study investigating macrophage cell lines, a hierarchical oxidative stress response was found (Xiao et al. 2003). The Nrf2 pathway, responsible for the transcription of antioxidant enzymes, responds to moderate levels of oxidative stress but beyond a threshold, high ROS concentrations overwhelm the antioxidant capacity resulting in the consumption of antioxidants and the downregulation of Nrf2 (Xiao et al. 2003; Myatt and Cui 2004; Faraonio et al. 2006; Liu et al. 2008). Chronically high levels of oxidative stress have been shown to directly inactivate antioxidant enzymes, reducing their overall concentrations, and impairing the ability to mount an antioxidant defense (Pigeolet et al. 1990; Sultana et al. 2017). Our study population differs from most oxidative stress studies as all participants were pregnant. Blood samples from previous studies have demonstrated that pregnant individuals have higher basal levels of oxidative stress and altered antioxidant activity (Wisdom et al. 1991; Morris et al. 1998; Llurba et al. 2004; Myatt and Cui 2004). These unique characteristics may affect how pregnant individuals respond to similar levels of exposure-induced oxidative stress compared with nonpregnant individuals.

In this study, SOD concentrations were found to decrease with increased proximity to UOG wells. This study is not the first to observe decreases in SOD in a population exposed to oxidative stress-inducing pollutants. Karagözler et al. (2002) observed significantly lower blood concentrations of SOD and a 2-fold increase in blood CAT concentrations in response to chronic VOC exposure in house painters. Hormozi et al. (2018) performed a similar study examining the effects of lead and cadmium exposure on antioxidant enzymes in tile glazers. Statistically significant decreases in SOD and TAC were found alongside increases in CAT in blood samples (Hormozi et al. 2018). Both studies noted that explanatory mechanisms were difficult to pinpoint, but they postulate that decreased SOD may be a result of VOC-induced enzyme inhibition or depletion of substrate molecules (Karagözler et al. 2002). This is supported by Pigeolet et al. (1990) who found that high ROS concentrations can result in the direct inactivation of antioxidant enzymes. Studies examining epithelial and cardiac cells have previously found a separate mitochondrial CAT production pathway, potentially explaining CAT increases in previous studies and the increasing CAT trends with proximity to oil and gas wells in the present study when other antioxidant enzyme concentrations are decreased (Bai and Cederbaum 2001; Rindler et al. 2013).

The observed decrease in SOD may also be driven by a stress-induced decrease in aMT6s. Melatonin is an activator of the Nrf2 antioxidant pathway which transcribes antioxidant enzymes (Ahmadi and Ashrafizadeh 2020; Shaw and Chattopadhyay 2020; Chen et al. 2022). Decreased melatonin would therefore result in reduced transcription of antioxidant enzymes. Urinary aMT6s correlates strongly with blood melatonin concentrations (Bojkowski et al. 1987; Graham et al. 1998; Braam and Spruyt 2022), so it is reasonable to assume that the observed decreasing trend in aMT6s in response to elevated proximity to wells corresponds with a decrease in blood melatonin concentrations. This decrease in blood melatonin may be stress-induced as populations living near oil and gas wells have previously reported increased stress due to noise, light pollution, environmental pollution, traffic, and stressed community infrastructures (Elliott et al. 2018; Malin 2020; Aker et al. 2022). Increased perceived stress has previously been linked to increased blood cortisol levels (van Eck and Nicolson 1994; Van Uum et al. 2008; Bozovic et al. 2013; Etwel et al. 2014). Melatonin and cortisol are inversely related in the human body (Fei et al. 2004; Claustrat et al. 2005) and murine models have shown cortisol-mediated stress-induced inhibition of melatonin synthesis in the pineal gland (López-Patiño et al. 2014). This pathway may explain the observed decrease in melatonin in participants living near oil and gas wells but more research investigating melatonin-cortisol interactions in humans will be needed before drawing conclusions.

The results from both mechanistic and real-world studies may indicate that, up to a threshold, oxidative stress activates the antioxidant response, but chronically high levels of oxidative stress may overwhelm the antioxidant response resulting in a decrease in antioxidant activity through the activation of other protective pathways, the consumption of antioxidants, and the direct inactivation of antioxidant enzymes. Decreases in SOD may also be driven by stress-induced decreases in blood melatonin due to proximity to stress-inducing oil and gas sites.

### Oxidative stress

Although no associations were found between oil and gas proximity metrics and biomarkers of lipid peroxidation, MDA, and 8-IP, there were associations found with some indoor air VOC concentrations. Hexanal was significantly associated to both an increase in 8-IP and a decrease in aMT6s. Both hexanal and 8-IP are byproducts of lipid peroxidation (Janicka et al. 2010; Elisia and Kitts 2011) so it is not surprising to find some association between the 2. However, although hexanal has been measured in exhaled breath, indoor air hexanal concentrations are often significantly higher than what can be accounted for by human respiration alone, indicating a fraction coming from exogenous sources (Mochalski et al. 2013). An analysis of hexanal source attribution could help determine the impacts of endogenous vs. exogenous indoor air hexanal concentrations on urinary 8-IP concentrations. Increased indoor air concentrations of 1,4-dioxane were associated with a decrease in MDA. 1,4-dioxane, which has previously been identified in flowback water from UOG activity (Lester et al. 2015), is classified as a probable carcinogen (Godri Pollitt et al. 2019) and has been shown to increase oxidative stress (Kurt et al. 2021). MDA would therefore be expected to increase in association with 1,4-dioxane exposure, but the opposite was observed.

Our analysis found that 8-OHdG significantly decreased with elevated density and proximity of all wells and UOG wells. This contradicts other studies which find increases in 8-OHdG with elevated exposure to VOCs (Chuang et al. 2007; Lu et al. 2007; Qian et al. 2021). One possible explanation may lie in the mechanisms of DNA repair. When DNA is oxidized, 8-OHdG is formed within the DNA strand and upon repair, 8-OHdG is released (Halliwell 2000). Without DNA repair, 8-OHdG is not released despite potential DNA damage. Melatonin plays a key role in repairing DNA segments and may affect the urinary excretion of 8-OHdG (Morioka et al. 1999). A previous study demonstrated positive correlations between 8-OHdG and aMT6s concentrations (He et al. 2020), matching our results. Bhatti et al. (2017) investigated melatonin-deficient night-shift workers and found that decreases in 8-OHdG mirrored decreases in melatonin. If our participants do indeed have lower aMT6s concentrations due to the increased stress associated with living near oil and gas wells, the association between urinary aMT6s and 8-OHdG concentrations shown in the current literature may explain why this study found a decrease in urinary 8-OHdG in response to increased exposure where other studies found the opposite.

### Limitations

There are several limitations affecting the robustness of these findings. Previous studies have shown that uric acid concentrations can influence TAC assay kit results (Karamouzis et al. 2008; Benkhai et al. 2010). Uric acid was not measured or included as a covariable in this analysis. Small sample size also weakened statistical power, potentially masking significant trends. Additionally, uncontrolled variables like time spent indoors could attenuate the relationship between biomarker concentrations and indoor air VOC concentrations.

Every effort was made to minimize freeze-thaw cycles but even so, samples experienced 2 to 3 freeze-thaw cycles by time of measurement. Lastly, this study ran 288 statistical tests resulting in 15 (5.2%) statistically significant results. This is approximately what would be expected from chance findings with *P*-values of 0.05.

## Conclusion

Some associations were found between oil and gas wells metrics, indoor air VOC concentrations, and altered biomarker concentrations of oxidative stress and antioxidant activity. Among these associations, proximity to oil and gas wells was associated with decreasing urinary concentrations of SOD, aMT6s, and 8-OHdG. Indoor hexanal concentrations were associated with 8-IP. Indoor 1,4-dioxane concentrations were negatively associated with MDA, contradicting current literature. Although associations were found linking proximity to oil and gas wells to altered oxidative stress and antioxidant activity, the degree to which UOG activity impacts oxidative stress and antioxidant activity remains unclear. These findings along with growing epidemiological evidence linking UOG exposure and negative birth outcomes reinforce the need to assess the health risks associated with this industry, especially in vulnerable populations like pregnant individuals.

## Data Availability

We will not make additional data for EXPERIVA participants available to external investigators given restrictions in our research agreements and ethics approval.
